# Epidemiology and clinical management of 1072 dogs with diabetes mellitus in a UK diabetes register

**DOI:** 10.1186/s40575-025-00146-x

**Published:** 2025-10-10

**Authors:** A. L. Denyer, D. G. O’Neill, D. C. Brodbelt, A. Holder, B. Catchpole, L. J. Davison

**Affiliations:** 1 Pathobiology and Population Sciences, The Royal Veterinary College, Hawkshead Lane, North Mymms, Hatfield, AL9 7TA Herts UK; 2 Department of Clinical Science and Services, Royal Veterinary College, Hatfield, UK; 3 Department of Physiology, Anatomy and Genetics, University of Oxford, Oxford, UK

**Keywords:** Dog, Diabetes, Veterinary, Epidemiology, VetCompass, Breed

## Abstract

**Background:**

The UK Canine Diabetes Register and Archive (UKCDRA), established in 1999, is a valuable resource of clinical data and blood samples from diabetic dogs. This study aimed to provide updated information about the epidemiology and clinical management of canine diabetes mellitus (DM) in UK veterinary practices.

**Method:**

Data from samples submitted to UKCDRA between December 2005 and December 2019 were divided into three groups according to DM aetiology: juvenile-onset, adult-onset non-dioestrus and adult-onset female entire. Epidemiological and clinical factors were analysed across groups. Breeds with more than 5 UKCDRA DM cases and/or ≥ 5000 dogs in the non-diabetic VetCompass denominator population were compared to those in the wider UK VetCompass™ population to explore breed DM risk.

**Results:**

Ninety-nine breeds were represented in the study, with 25 breeds having 5 or more diabetic cases. Samoyeds and Tibetan terriers demonstrated the highest odds of DM in both of the adult-onset DM groups. Age of adult-onset DM onset co-varied with breed, with the Standard Doberman Pinscher and Rottweiler demonstrating youngest onset age. Twice daily Caninsulin (40 iu/ml porcine insulin) was the most commonly reported treatment. Twice-daily rather than once-daily insulin therapy in canine DM has become more prevalent since the archive was founded.

**Limitations:**

UKCDRA does not capture detailed information on concurrent diseases or DM environmental risk factors.

**Conclusion:**

This study provides an update to an earlier UKCDRA report and demonstrates shared breed-associated risk factors for adult-onset non-dioestrus and female entire DM.

**Supplementary Information:**

The online version contains supplementary material available at 10.1186/s40575-025-00146-x.

## Introduction

Diabetes mellitus (DM) is a clinical syndrome of hyperglycaemia due to inadequate secretion and/or action of insulin from the pancreas [1]. Criteria for diagnosis include a blood glucose concentration of > 11.1 mmol/l (> 200 mg/dl) with classic clinical signs of hyperglycaemia, or confirmation of persistent hyperglycaemia by measurement of glycated blood proteins such as fructosamine [1]. Canine DM is estimated to occur in 0.26–1.33% of dogs [2–7] and will be encountered regularly by most veterinarians in companion animal practice. Affected dogs usually require lifelong insulin treatment for survival [8, 9] placing a significant burden on owners and requiring regular and lifelong veterinary care. Studying the disease epidemiology and current management is valuable to improve understanding of the subsets of dogs at greatest risk and to identify clinical benchmarks to contribute to improved management.

The UK Canine Diabetes Register and Archive (UKCDRA) is a large database of clinical information relating to diabetic dogs in the UK and an archive of associated blood samples submitted for evaluation of glycaemic control. The archive was established in 1999 for the purpose of research into canine DM. An early study using the samples received between 2000 and 2003 made some important observations about breed risk of DM and the relationship of insulin treatments to measures of glycaemic control [4]. An update to the breed distribution of diabetic dogs in the UKCDRA was published in 2013 [10], describing archive submissions between 2000 and 2010. In more than 20 years since the archive began, over 2000 samples have been submitted, alongside corresponding clinical data. These samples and data have underpinned a large number of studies of canine DM and continue to do so [11, 12].

Canine DM is a complex disease caused by various combinations of genetic and environmental factors [11, 13 and 14]. Although rare cases are reported in dogs aged under 12 months, DM typically develops between 5 and 12 years of age [2, 4]. There is currently no universally accepted diagnostic classification, but insulin dependent and insulin resistant forms of canine DM have been described, [15] evolving into the most recent publication and classification system on this topic by Project ALIVE (Agreeing Language in Veterinary Endocrinology) [1]. Most canine diabetic patients are dependent on external insulin injections [4] and the reduced numbers of beta cells observed in the pancreas of diabetic dogs [16, 17] suggest that canine DM results from beta cell loss and /or failure of regeneration. In some dogs, insulin deficiency may be preceded by insulin resistance, which can be due to the effects of drugs or hormones on the action of insulin [1]. For example, dioestrus-associated diabetes is recognised in entire female dogs associated with the insulin antagonistic action of growth hormone released from the canine mammary glands during the progesterone-dominated phase of dioestrus, and gestational diabetes can also occur in canine pregnancy [18, 19]. Similarly, exogeneous and endogenous corticosteroids antagonise the action of insulin, which may lead to persistent hyperglycaemia and eventually DM [1]. Being overweight or obese is also a reported risk factor in dogs [6, 7, 20] although a causal link has not been established and most obese dogs appear to compensate appropriately by increasing insulin secretion [21]. Therefore, canine DM is a highly heterogeneous disease [13] and more work is required to improve classification and facilitate tailored management of patients.

Within the canine diabetic population, some breeds are much more highly represented than others. Breeds such as the Australian Terrier, Samoyed and Miniature Schnauzer are reported to be at increased risk in multiple studies [5, 7, 22] whereas the Boxer, Golden Retriever and German Shepherd Dog are consistently reported to be at lower risk [4, 6, 22]. Juvenile forms of canine DM have also been reported to occur particularly in certain breeds, such as Keeshonds [23] and Labrador Retrievers [10, 24–25]. These apparent breed predispositions strongly suggest a genetic component to the aetiology of canine DM, acting in combination with environmental factors. The association of genetic variants with DM in candidate gene studies in specific breeds further supports this suggestion [26–29]. As well as providing important health information for veterinary surgeons, dog owners and breeders, identifying breeds at higher or lower risk of disease can help to inform the design of studies to identify genetic variants associated with differential risk of DM [12].

While insulin injections are the mainstay of treatment of canine DM, the insulin preparation and dosing schedule is usually determined on an individual dog basis and may be modified in response to clinical signs and other monitoring tools [9]. The availability and popularity of different insulin preparations also changes over time. There are currently 2 licensed veterinary insulin products in the UK: Caninsulin (MSD Animal Health) is a mixed soluble and lente form of porcine zinc insulin; ProZinc (Boehringer Ingelheim Animal Health UK Ltd) is a longer acting protamine zinc recombinant human insulin. Caninsulin was licensed prior to the establishment of the archive, however ProZinc was not marketed until 2016, and initially was only licensed for use in cats. When the archive was established, a bovine insulin, Insuvet (Schering Plough Animal Health), was also available and licensed in neutral (soluble), lente and protamine zinc (PZI) formulations, but these preparations were discontinued in 2010.

Monitoring of glycaemic control is essential to determine the appropriate insulin dose and frequency of administration (once or twice daily). Alongside careful evaluation of clinical signs, blood glucose curves and continuous interstitial glucose monitoring are also useful to indicate daily blood glucose fluctuations and response to insulin. In addition, measurement of the concentration of fructosamine and glycated haemoglobin can be helpful to indicate glycaemic control over a longer period (1–2 week and 2–3 months respectively) [9, 30].

The purpose of the present study is to provide an update to the first report from the UKCDRA, published in 2005 [4]. The study aims to identify trends in canine DM management in UK primary care veterinary practices and to understand breed susceptibility by comparing the frequency of breeds in the diabetes archive to breeds represented in a large database of UK primary care veterinary practices: VetCompass [31].

## Methods

### Sample population

Blood samples taken from UK diabetic dogs for diagnostic or monitoring purposes were submitted by first opinion and referral veterinary practices to the UKCDRA at the Royal Veterinary College (RVC) in response to advertising in the veterinary press. This was a voluntary submission process, with samples being submitted by post and processed on the day of arrival in the laboratory. DM had been diagnosed by submitting practitioners on the basis of clinical signs, persistent hyperglycaemia, glucosuria and / or elevated fructosamine concentration. Submitting practitioners were asked to provide information about age, sex, neuter status, breed, bodyweight and concurrent conditions and treatments using a sample submission form. Information about date of diagnosis of diabetes and insulin treatment type and dose at time of submission were also requested. Fructosamine and glycated haemoglobin measurements were carried out and results returned to the submitting practice free of charge. Residual blood was stored frozen. Case information submitted alongside samples was stored in accordance with relevant data protection legislation. Data and blood samples were collected between 1999 and 2019 before collection was discontinued temporarily in early 2020, during the Covid-19 pandemic. From March 2004, submitting practitioners were also asked to provide information about date of neutering (in addition to date of DM diagnosis) for female neutered cases. Ethical approval for the UKDRA was obtained from the RVC Ethics and Welfare Committee and renewed on a 5-yearly basis (approval number at the time of the current analysis: URN 2017/1685-3).

### Fructosamine and glycated haemoglobin

Prior to September 2003, and in the first report of the UKCDRA, fructosamine was measured by Vetlab Services Ltd. (Horsham, UK). After this date, fructosamine was measured by RVC Diagnostic Laboratory Service (kinetic assay by Catachem inc, USA; reference range 184-399umol/L). Glycated haemoglobin was initially measured using a Haemaquant device and disposable Glycosal test cartridges (Provalis), but from December 2005 the A1CNOW test kit (BHR pharmaceuticals) was used [32]. In order to ensure consistency of techniques for clinical measurements, and because findings from the early years of the archive have previously been published, only cases submitted after December 2005 were included in the analysis described below.

### Analysis groups

Cases submitted to the archive between December 2005 and December 2019 were selected for the current analysis and were further sub-grouped according to suspected aetiology of DM (Table 1). Group 1 comprised cases with juvenile-onset DM, diagnosed ≤ 1 year of age, of all sex-neuter categories. The remaining two groups consisted of cases diagnosed ≥ 3 years of age, so as to include only cases with an adult-onset form of the disease and avoid overlap with juvenile-onset diabetes. Group 2 included adult-onset male and female cases with a non-dioestrus aetiology: this comprised male entire diabetic dogs and female/male cases reported to have been neutered before diagnosis. Group 3 comprised cases recorded as adult-onset entire female at time of diagnosis; these were considered most likely to have dioestrus-induced DM.

### VetCompass denominator population for calculation of breed risk of DM

A case-control study design was undertaken to explore the odds ratio for DM among a range of dog breeds in the UK. Diabetic cases of each breed aged ≥ 3 years at diagnosis in the UKCDRA were compared to control (non-diabetic) dogs derived from the largest available UK reference population of dogs under primary veterinary care: VetCompass. VetCompass collates de-identified electronic health record (EHR) data from primary-care veterinary practices in the UK for epidemiological research [31]. The method of using this external national dataset as a comparator for demographic comparisons and analysis has been employed previously in studies of other canine conditions [33–35]. The VetCompass population included all available dogs aged ≥ 3 years under primary veterinary care at clinics participating in the VetCompass Programme during 2016. Dogs under veterinary care were defined as those with either at least one EHR (VeNom Veterinary Nomenclature^1^ diagnosis term, free-text clinical note, treatment or bodyweight) recorded during 2016 or at least one EHR recorded during both 2015 and 2017. Relevant data fields available to VetCompass researchers include a unique animal identifier along with veterinary clinic identifier, species, breed, date of birth, sex and neuter along with clinical information from free-form text clinical notes, summary diagnosis terms (VeNom) and treatment with relevant dates. Ethical approval for the use of VetCompass data in the current study was obtained from the RVC Ethics and Welfare Committee (reference number SR2018-1652).

Dogs with increased probability of DM were excluded from the VetCompass control data. All dogs in the VetCompass denominator dataset up to December 31, 2016 were screened in the clinical free-text field using search terms diab*, insul*, mell*, and the treatments field using the search term insul*, canins*, insuv*, diab*. All dogs with at least one of these search terms were removed from the overall VetCompass population to generate a list of VetCompass dogs with no recorded evidence of DM that to be included as controls in the breed risk analysis.

### Statistical analysis – UKCDRA data

**Fig. 1:**
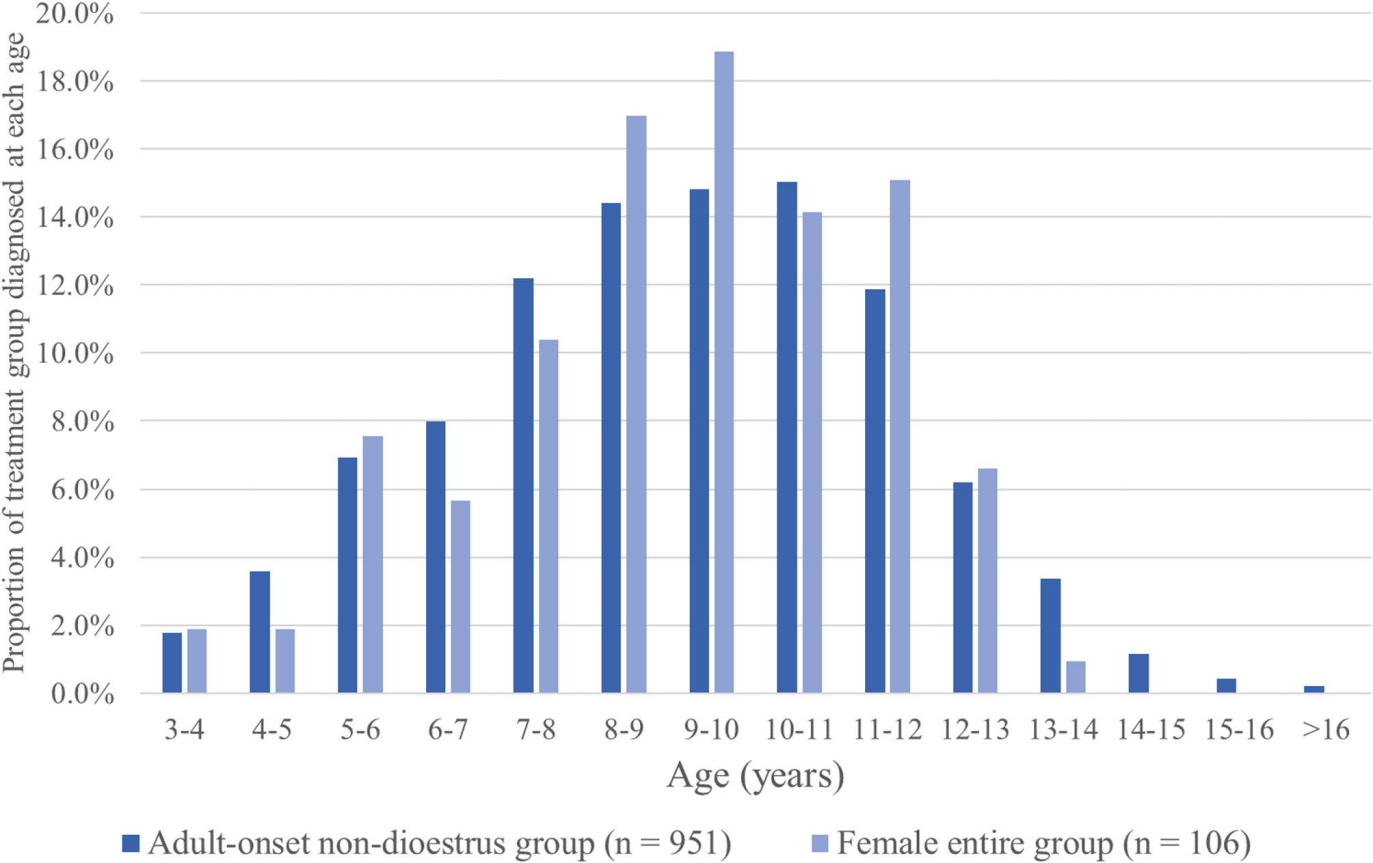
Proportion of adult-onset non-dioestrus DM cases (Group 2; *n* = 951 : male entire, male neutered and female neutered at diagnosis) and female entire DM cases (Group 3; *n* = 106) diagnosed at each age (years) between 3 and 16 years

**Fig. 2:**
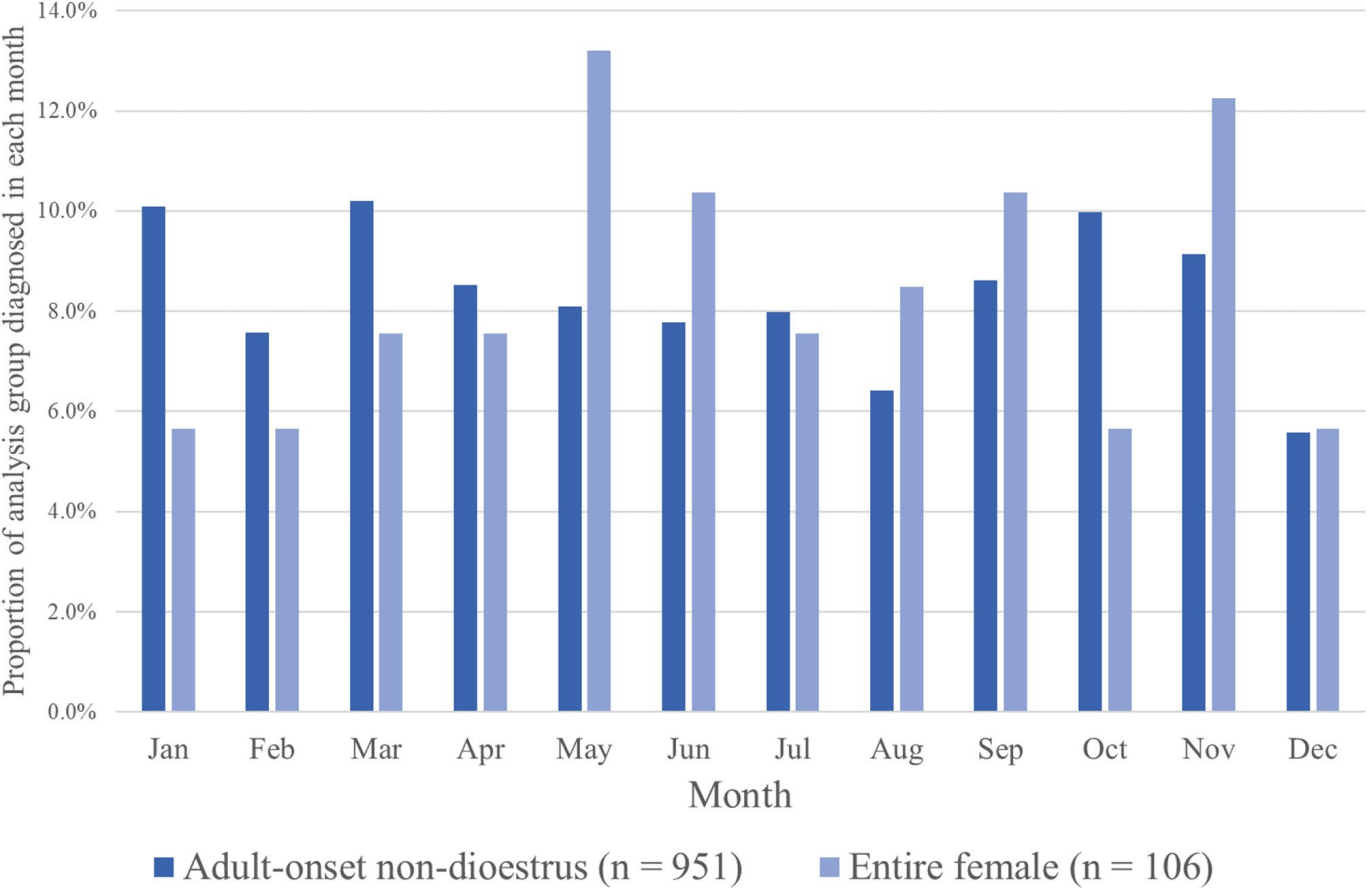
Proportion of adult-onset non-dioestrus DM cases (*n* = 951) and female entire DM cases (*n* = 106) aged ≥3 years diagnosed in each month of the year between December 2005 and December 2019

**Fig. 3:**
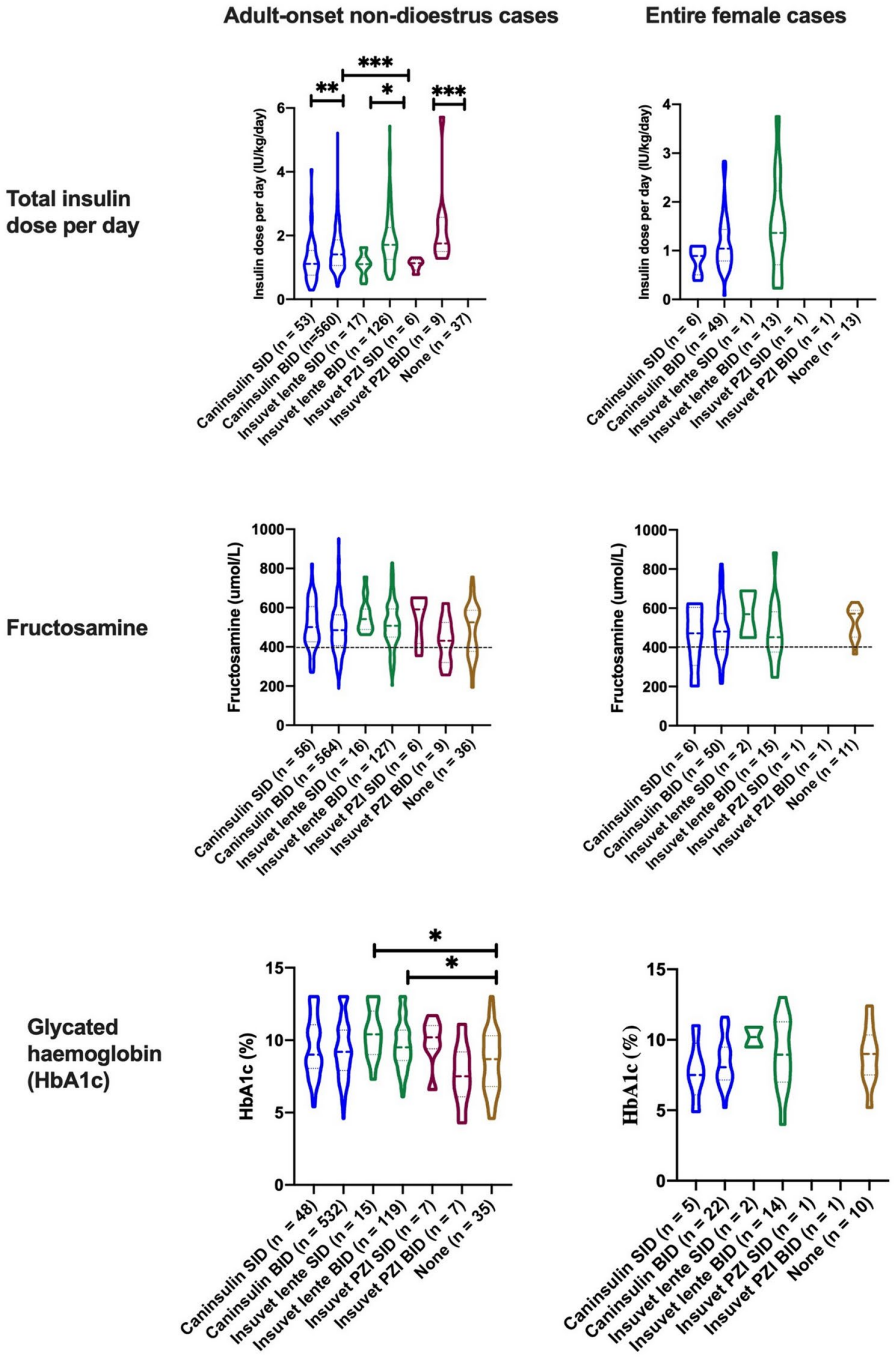
Violin plots displaying distribution of clinical variables (daily insulin dose and concentrations of fructosamine and glycated haemoglobin) among adult-onset DM cases treated with different insulin preparations for at least one month or not receiving insulin treatment. Significant differences between treatment groups are denoted by * *p* < 0.05, ** *p* < 0.005, *** *p* < 0.0005. Dashed line indicates upper limit of reference range for fructosamine (399umol/l). Treatment groups with single cases are not shown. SID once a day, BID twice a day

**Fig. 4:**
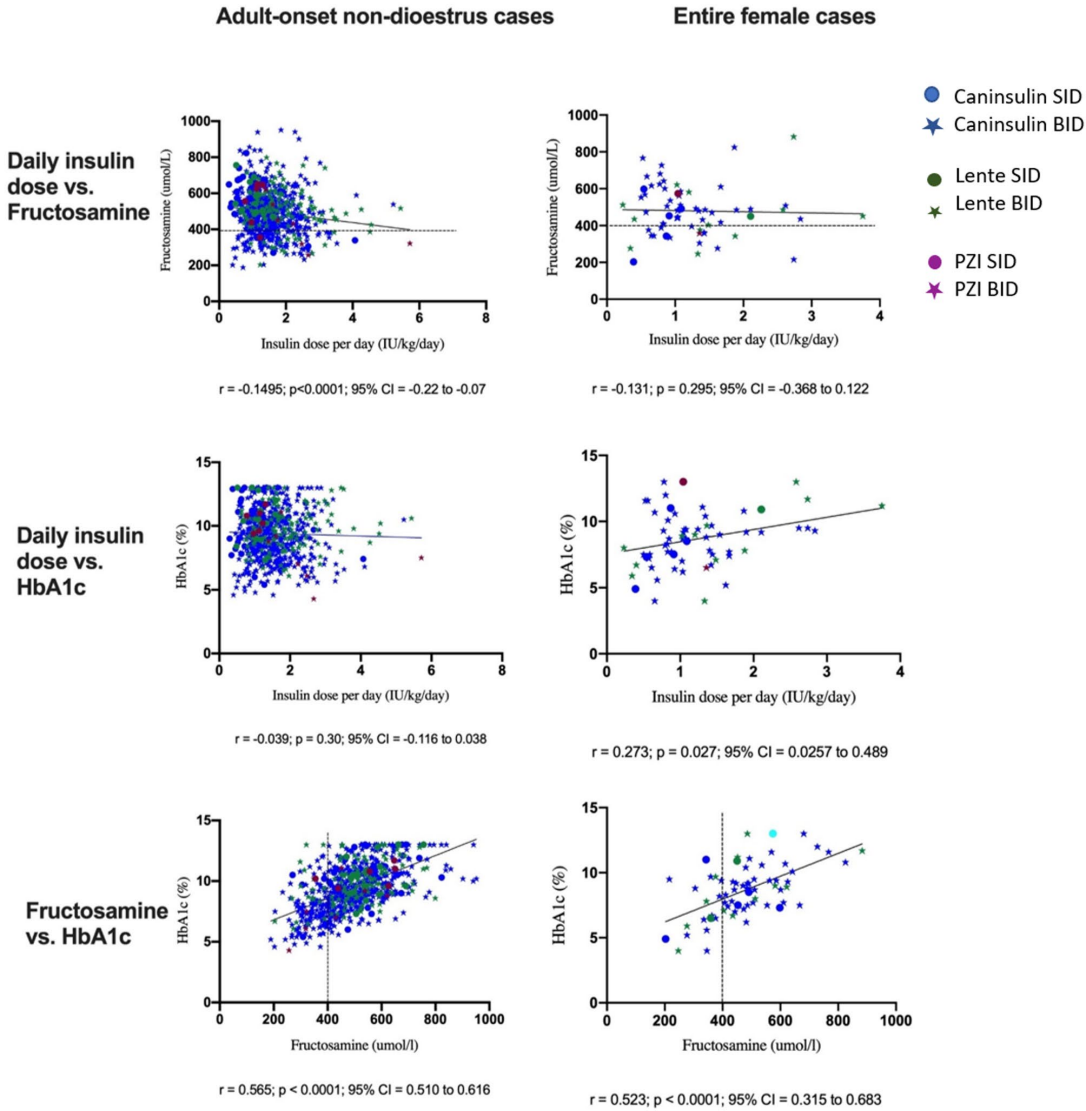
Correlation between clinical variables in cases treated with insulin for at least one month. Colours indicate different insulin treatments and shapes indicate frequency of treatment. Dashed line indicates upper limit of reference range for fructosamine. Note that the maximum HbA1c value recorded by the A1cNow device is 13.0%. Linear regression lines and results of Spearman rank correlation test are shown

**Fig. 5:**
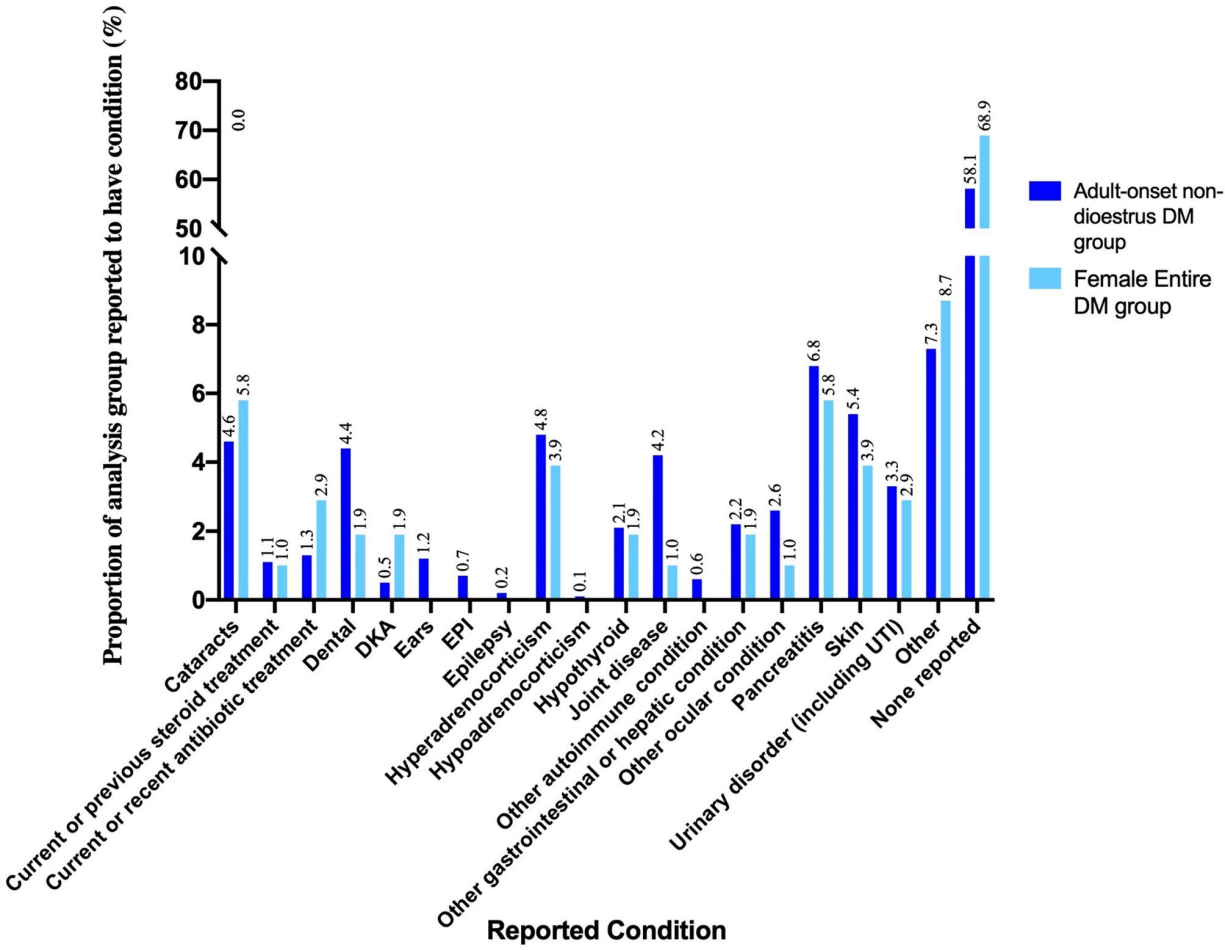
Proportion of concurrent conditions reported among adult-onset non-dioestrus DM and female entire DM analysis groups between December 2005 and December 2019. Includes confirmed and suspected cases of each condition listed. Key: Dental, dental disease; DKA, diabetic ketoacidosis; Ears, external ear disease; EPI, exocrine pancreatic insufficiency; UTI, urinary tract infection. Note that ‘None reported’ does not mean that the condition was not present, only that it was not reported on the sample submission form

**Fig. 6:**
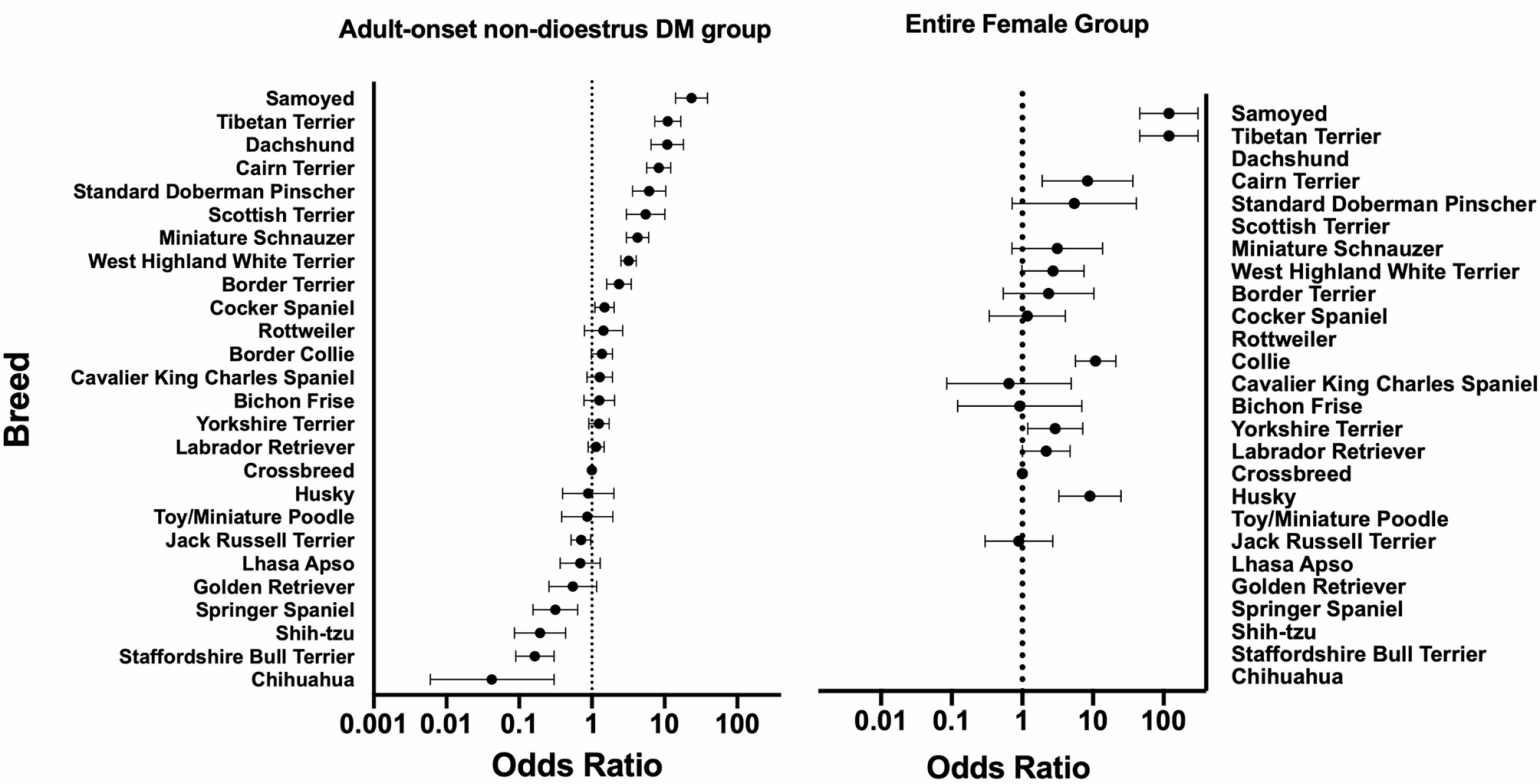
Forest plot illustrating odds ratio and confidence interval for breed risk of diabetes mellitus (DM) compared to Crossbreeds (Data given in Supplementary Tables 1 and 2). The number of dogs of each breed in the UKCDRA for Groups 2 and 3 in this study was compared to the total VetCompass denominator population (note that this included both male and female dogs of all neuter status within each breed). Breed risk was only calculated for breeds with > 5 cases in the UKCDRA and > 5000 controls in the VetCompass population. Vertical dashed line indicates odds ratio of 1.0. Note logarithmic scale on x axis

Data were cleaned and checked in Microsoft Office Excel (Microsoft Corp.) before exporting to GraphPad Prism 8 (GraphPad Software, LLC) and SPSS Statistics (IBM) for analysis. Charts were plotted in Microsoft Office Excel (Figs. 1 and 2) and GraphPad Prism 8 (Figs. 3, 4, 5 and 6).

Descriptive data are reported as Mean (Standard Deviation) if normally distributed and as Median (Inter-quartile range and range) if not normally distributed. Kruskal-Wallis test and Dunn’s multiple comparison test were used to compare clinical variables (duration of treatment; daily insulin dose; fructosamine concentration; HbA1c concentration) among insulin treatment groups. Spearman’s rank correlation test was used to analyse correlations between clinical variables; linear regression lines were added to each chart. One-way ANOVA was used to compare the mean age of diagnosis among breeds in the adult-onset non-dioestrus DM group in the UKCDRA (Group 2). Dunnett’s multiple comparison test was carried out with Crossbreed as the reference population.

### Statistical analysis – comparison of breeds in UKCDRA and VetCompass data

Multivariable binary logistic regression modelling was used to estimate the odds ratio for DM among different breeds compared to Crossbreeds. This was carried out separately for the adult-onset non-dioestrus group, including both males and females (Group 2) and the adult-onset female entire group (Group 3). Breeds included in this analysis were those with ≥ 5 cases in the adult-onset non-dioestrus group in the UKCDRA dataset and/or ≥ 5000 dogs in the non-diabetic VetCompass denominator population. Age of diabetes onset in the UKCDRA dogs was included as a continuous variable covariate in the model (adult-onset non-dioestrus group only), with all dogs in the model being ≥ 3 years of age. It was not possible to include sex or neuter status as covariates, due to the specific inclusion of particular sex-neuter categories in each of the UKCDRA analysis groups. Data relating to concurrent diseases also could not be included in the model because these data were not consistently provided for all UKCDRA cases and were unavailable for the wider VetCompass population. Statistical significance was set at the 5% level.

## Results

There were 1,072 DM cases aged ≤ 1years or ≥ 3 years at diagnosis in the UKCDRA, submitted between December 2005 and December 2019. Of these, 15 (1.4%) cases met criteria for inclusion in Group 1 (juvenile DM cases), 951 (88.7%) cases were included in Group 2 (adult-onset non-dioestrus DM cases – including dogs who were male, male neutered, or female neutered at diagnosis) and 106 (9.9%) cases were included in Group 3 (female entire DM cases) (Table 1). An additional 19 submitted cases were aged between 1 year and 3 years at the time of DM diagnosis so were not considered further in the analysis.

**Table 1:** Diabetic cases aged ≤ 1years or ≥ 3 years at diagnosis submitted to the UK canine diabetes register and archive (UKCDRA) between December 2005 and December 2019 divided into analysis groups according to known distinct aetiologies of canine DM. Number and characteristics of cases in each analysis group are shown

Analysis group		Age at diagnosis (years)	Recorded sex at diagnosis	Number of dogs	Median age at diagnosis (years)	IQR* age at diagnosis (years)	Range age at diagnosis (years; min – max)
1	Juvenile	≤ 1	FE, FN, ME, MN	15	0.3	0.2–0.6	0.1-1.0
2	Adult-onset non-dioestrus (entire and neutered male and neutered female)	≥ 3	FN, MN, ME	951	9.2	7.4–10.9	3.0–16.7
3	Entire female	≥ 3	FE	106	9.6	7.8–10.9	3.3–13.5

*(IQR, interquartile range; FN, female neutered; FE, female entire; MN, male neutered; ME, male entire)

In the VetCompass denominator population, from an available population of 905,543 dogs under veterinary care at 886 veterinary clinics during 2016, there were 9,898 (1.09%) dogs considered to have increased probability of DM based on the presence of one or more of the DM search terms in their clinical records. Following removal of these dogs, the remaining 895,645 dogs with no evidence of DM were included as the controls for DM. Following exclusion of dogs aged < 3 years on December 31, 2016, there were 749,827 dogs remaining in the VetCompass denominator population for comparative analysis.

### Annual submissions to UKCDRA

The number of samples submitted in each year of the archive was highly variable, dependent on a range of factors including staffing, funding and advertising. The median number submitted each year was 53 cases, interquartile range [IQR] 38.5–76, range 6–453), and samples were received from 161 UK unique practice postcodes.

### Juvenile group

Of the 15 submitted cases diagnosed at or below one year of age (Group 1), the median age of diagnosis was 0.31 years (Table 1). There were 8 (56%) male and 6 (40%) female cases (one unrecorded sex). The Labrador Retriever was the most highly represented breed (4 cases; 26.7%). There were two Crossbreeds (13.3%; Jack Russell Terrier cross, Dachshund cross) and nine remaining breeds, each with a single case (6.7%; Bernese Mountain Dog, Bichon Frise, Border Collie, Dachshund, Labradoodle, Standard Poodle, Whippet, West Highland White Terrier, Yorkshire Terrier).

Most were receiving Caninsulin (10 cases; 66%) with the remainder receiving Insuvet lente (2 cases; 13.3%) or no treatment at the time of submission (3 cases; 20%). The median dose of insulin for cases receiving treatment with data provided (*n* = 10) was 0.52 iu/kg/injection (range 0.22–1.25 iu/kg/injection).

### Adult-onset non-dioestrus group (neutered females, entire males, neutered males)

There were 951 cases in Group 2, aged ≥3 years at time of diagnosis at the submitting veterinary practice that were not entire female when first diagnosed with DM, but instead were male entire, male neutered or female neutered. Considering all breeds together, the median age at diagnosis was 9.2 years (IQR: 7.43–10.9; range: 3.0–16.7) (Table 1; Fig. 1). The mean age at diagnosis of DM was examined individually by breed in the adult-onset non-dioestrus DM group, but this analysis was only performed for breeds with > 5 diabetic dogs represented. The mean age at diagnosis of DM was lowest for Standard Doberman Pinscher (6.83 years) and greatest for Border Terrier (11.24 years) (Table 2). One-way ANOVA analysis of breeds identified a significant difference in the mean age of diagnosis among breeds (*p* value < 0.001). As a follow-up to one-way ANOVA, Dunnett’s test identified a significant difference between the mean age of diagnosis of DM in Crossbreeds (reference population) and that of the Standard Doberman Pinscher, Rottweiler, Cavalier King Charles Spaniel, Dachshund and Labrador Retriever breeds, which were all younger at the time of diagnosis than Crossbreeds (Table 2).

**Table 2:** Mean age of diagnosis of diabetes mellitus (DM) among breeds in the UK canine diabetes register and archive (UKCDRA) with > 5 dogs in the adult-onset non-dioestrus DM analysis group, containing dogs over the age of 3 years and reported to be female neutered, male neutered or male entire at DM diagnosis. A significant difference in mean age at diagnosis was identified by one-way ANOVA (*p* value < 0.0001). The results of Dunnett’s multiple comparison test following one-way ANOVA are shown. Asterisks (*) indicate a significant difference between a breed and the reference population (Crossbreeds). ^π^CI confidence interval

				Dunnett’s Multiple Comparison test			
Breed	Number of dogs	Mean age of diagnosis of DM (years)	Standard Error of Mean	Difference in mean age of diagnosis (years) of DM compared to reference (Crossbreed)	95% CI^π^ of Difference	Adjusted *P* Value	
Standard Doberman Pinscher	15	6.83	0.51	2.90	0.95–4.83	< 0.001	***
Rottweiler	11	7.04	0.75	2.68	0.44–4.93	0.006	**
Husky	6	7.27	1.12	2.45	-0.56–5.46	0.248	
Cavalier King Charles Spaniel	27	7.62	0.36	2.10	0.62–3.58	< 0.001	***
Dachshund	16	7.74	0.53	1.98	0.10–3.86	0.030	*
Scottish Terrier	11	8.36	0.48	1.36	-0.89–3.6	0.762	
Springer Spaniel	8	8.39	0.85	1.34	-1.28–3.95	0.934	
Samoyed	17	8.51	0.68	1.21	-0.62–3.04	0.620	
Tibetan Terrier	26	8.52	0.51	1.21	-0.3–2.71	0.278	
Labrador Retriever	83	8.62	0.23	1.11	0.17–2.04	0.008	**
Cairn Terrier	31	8.63	0.48	1.09	-0.31–2.49	0.315	
Miniature Schnauzer	36	8.81	0.36	0.91	-0.39–2.2	0.516	
Yorkshire Terrier	45	8.81	0.41	0.90	-0.28–2.1	0.352	
Cocker Spaniel	52	8.99	0.33	0.73	-0.39–1.85	0.644	
Shih-tzu	6	9.11	1.01	0.61	-2.4–3.61	0.999	
Golden Retriever	7	9.58	0.60	0.14	-2.65–2.93	> 0.999	
Border Collie	42	9.71	0.34	0.01	-1.21–1.24	> 0.999	
**Crossbreed**	**215**	**9.72**	**0.17**	**Reference**	**n/a**	**n/a**	
Jack Russell Terrier	49	9.78	0.33	-0.06	-1.21–1.09	> 0.999	
West Highland White Terrier	95	9.82	0.23	-0.10	-0.99–0.8	> 0.999	
Bichon Frise	18	9.85	0.75	-0.13	-1.91–1.65	> 0.999	
Staffordshire Bull Terrier	11	10.22	0.55	-0.50	-2.74–1.75	0.999	
Lhasa Apso	10	10.92	0.83	-1.20	-3.55–1.15	0.935	
Border Terrier	11	11.24	0.38	-1.52	-3.77–0.72	0.571	

Using the reported date of diagnosis, the proportion of cases diagnosed with DM in each month showed no clear pattern of seasonality, although notably fewer cases were diagnosed in December (Fig. 2).

Table 3 shows the number of cases treated with different insulin preparations, either once a day (SID) or twice a day (BID), for at least one month, as well as the median, inter-quartile range (IQR) and range of the duration of treatment, daily insulin dose and concentration of fructosamine and glycated haemoglobin (HbA1c). Cases recorded as not receiving insulin treatment, usually newly diagnosed patients, were included as a separate treatment group (“None”). Those receiving insulin treatment but for less than one month were not included in analysis of fructosamine or HbA1c data, since results in the first month of therapy may also reflect a pre-diagnosis period of normoglycaemia and / or a period of profound hyperglycaemia prior to beginning treatment. It was not possible to calculate dose per kg in cases where no bodyweight value was provided. Similarly, cases with missing data for fructosamine concentration or glycated haemoglobin could not be included in the median and range calculations for these measurements. For all cases receiving insulin twice day, the daily total insulin dose was calculated using the sum of the morning and evening doses. In 33 cases, the insulin dose was different in the morning compared to the evening. Caninsulin BID was the largest treatment group (*n* = 582), followed by Insuvet lente BID (*n* = 131). The smallest groups were Insuvet lente SID (*n* = 17) and both Insuvet PZI groups (*n* = 7 SID; *n* = 9 BID).

For all insulin preparations, the median duration of treatment was greater in cases being treated twice a day than those treated once a day. The daily insulin dose differed significantly among insulin treatment groups (*p* < 0.001). For all treatments, the daily total dose was significantly higher in cases treated twice a day than those treated once a day (*p* < 0.05 comparing SID to BID for all insulin types), but for each type of insulin, the median daily total dose in BID groups was less than double the median daily dose in SID groups. The daily total dose was also significantly lower in the Caninsulin BID group compared to the Insuvet lente BID group (*p* = 0.0002) (Fig. 3).

There was a significant difference in fructosamine concentration among treatment groups (*p* = 0.03) although no treatment groups were significantly different compared to the group not receiving insulin treatment. There was also a significant difference in HbA1c concentration among treatment groups (*p* = 0.0046); the Insuvet lente SID and Insuvet lente BID groups had significantly higher HbA1c concentration compared to the group not receiving insulin treatment (*p* = 0.011 and *p* = 0.018 respectively), potentially reflecting a period of normoglycaemia pre-diagnosis period in the untreated cases (Fig. 3). The daily insulin dose was weakly negatively correlated with fructosamine concentration when considering all treatment groups together (*r* = -0.1495, *p* < 0.0001) (Fig. 4) but was not correlated with HbA1c (*p* = 0.3). There was a strong positive correlation between fructosamine concentration and HbA1c when considering all treatment groups together (*r* = 0.565, *p* < 0.0001).

**Table 3:** Median (interquartile range; range) of clinical variables in the adult-onset non-dioestrus DM analysis group (Group 2) comprising dogs who were male entire, male neutered or female neutered at diagnosis, treated with different insulin preparations for > 1 month or untreated. SID once a day, BID twice a day

Insulin treatment	Number of dogs	Duration of treatment (months)	Total Daily insulin dose (iu/kg/day)	Fructosamine concentration (umol/l)	HbA1c (%)
None	37	NA	NA	525 (378–585; 194–756)	8.7 (6.85–10.25; 4.6–13)
Caninsulin SID	56	4.38 (1.75–10.8; 1.10–69.5)	1.11 (0.81–1.45; 0.29–4.07)	501 (430–599; 270–823)	9.0 (8.15–11.0; 5.4–13)
Caninsulin BID	582	9.48 (4.06–21.6; 1.00–81.37)	1.41 (1.06–1.86; 0.40–5.22)	486 (405–564; 189–952)	9.2 (7.9–10.7; 4.6–13)
Insuvet lente SID	17	3.57 (1.60–8.17; 1.03–29.47)	1.10 (0.92–1.30; 0.49–1.62)	542 (493–592; 463–756)	10.20 (9.3–11.6; 7.30–13)
Insuvet lente BID	131	12.13 (6.20–23.4;1.03–64.20)	1.71 (1.26–2.24; 0.63–5.43)	508 (450–594; 204–829)	9.5 (8.6–10.7; 6.1–13)
Insuvet PZI SID	7	6.77 (2.95–25.1; 1.03–33.27)	1.13 (1.01–1.20; 0.78–1.30)	591 (468–642; 355–650)	10.20 (9.5–10.9; 6.60–11.7)
Insuvet PZI BID	9	19.17 (4.37–26.0; 3.20–65.03)	1.75 (1.60–2.48; 1.28–5.71)	432 (322–513; 257–621)	7.50 (6.55–9; 4.30–11.1)

### Entire female group

There were 106 cases aged ≥3 years at time of diagnosis that were reported as entire female when first diagnosed with DM and considered likely to have dioestrus DM (Group 3). The median age at diagnosis in this group was 9.6 years and followed similar pattern of distribution to that of the adult-onset non-dioestrus group, although no cases > 14 years were present in the entire female group (Table 1; Fig. 1). There was a greater fluctuation in the proportion of cases diagnosed with DM in each month of the year compared to the adult-onset non-dioestrus group (Group 2); there was no clear seasonal pattern, although number of diagnoses tended to be greater in months May – September than in the winter months, excluding November (Fig. 2). There was no capacity to determine whether any diabetic dogs in this study entered diabetic remission following insulin therapy or neutering. However, this is considered unlikely as remission is an extremely rare event in canine diabetes mellitus, and the majority of patients were receiving long term (> 1 month) insulin therapy.

Caninsulin BID was, again, the largest treatment group, followed by Insuvet lente BID (Table 4). Only two cases were treated with Insuvet PZI (one SID and one BID); these are not shown in Fig. 3. There was no significant difference in daily insulin dose, fructosamine concentration or HbA1c among treatment groups. The daily insulin dose, fructosamine concentration and HbA1c were comparable to the respective values in the adult-onset non-dioestrus group (Table 4; Fig. 3).

The daily insulin dose was positively correlated with HbA1c by Spearman rank test, when considering all treatment groups together (*r* = 0.273, *p* = 0.027) (Fig. 4), but was not correlated with fructosamine concentration (*p* = 0.295). The correlation of fructosamine concentration with HbA1c was similar to the correlation in the adult-onset non-dioestrus group (*r* = 0.523, *p* < 0.0001).

**Table 4:** Median (interquartile range; range) of clinical variables in DM analysis group comprised of dogs that were female entire at the time of diagnosis (Group 3), treated with different insulin preparations for > 1 month or untreated. SID once a day, BID twice a day

Insulin treatment	Number of cases	Duration of treatment (months)	Total Daily insulin dose (iu/kg/day)	Fructosamine concentration (umol/l)	HbA1c (%)
None	13	NA	NA	571 (458–586; 365–629)	9.0 (7.6–9.9; 5.2–12.4)
Caninsulin SID	6	5.9 (3.48–33.3; 1.27–46.1)	0.89 (0.63–1.04; 0.39–1.09)	472 (31–571; 203–623)	7.5 (7.3–8.5; 4.9–11)
Caninsulin BID	51	5.87 (3.02–16.9; 1.13–60.1)	1.04 (0.80–1.43; 0.08–2.84)	481 (391–568; 216–825)	8.8 (7.5–9.55; 4–13)
Insuvet lente SID	2	1.00 (0.71–1.30; 0.41–1.59)	2.11	570 (50–629; 450–689)	10.2 (9.85–10.6; 9.5–10.9)
Insuvet lente BID	15	10.7 (5.42–16.6; 1.5–64)	1.36 (1.03–1.88; 0.23–3.75)	452 (390–559;247–883)	8.95 (7.28–11.1; 4–13)
Insuvet PZI SID	1	94.5	1.04	574	13
Insuvet PZI BID	1	41.1	1.36	359	6.5

### Concurrent diseases

The reporting of concurrent conditions and treatments as free text on the sample submission form was inconsistent across submissions. A variety of concurrent conditions and treatments were reported by submitting veterinarians, with a variable degree of detail and certainty. Figure 5 shows the proportion of the adult-onset non-dioestrus DM and female entire DM analysis groups reported to have each condition listed, including both confirmed and suspected cases. It was not possible to distinguish these accurately based on the information provided on the sample forms. The “none reported” group includes cases for which no information about concurrent diseases was provided, as well as cases reported specifically not to have any known concurrent conditions.

The most commonly reported individual concurrent conditions in the UKCDRA diabetic dogs were cataracts, pancreatitis, hyperadrenocorticism and skin conditions (Fig. 5). Sixty-three cases (6.62%) in the adult-onset non-dioestrus group (Group 2) and 5 cases (4.81%) in the female entire group (Group 3) were reported to have more than one concurrent condition.

### Breed and age associations with DM in the UKCDRA

There were 99 different dog breeds represented in the UKCDRA. Among the adult-onset non-dioestrus DM group (Group 2), comprising dogs who were male entire, male neutered or female neutered at diagnosis, the largest single breed group was Crossbreeds (24.86%, *n* = 215). The most highly represented individual breeds in samples collected since December 2005 were West Highland White Terriers (*n* = 95, 10.98%) followed by Labrador Retrievers (*n* = 83, 9.60%). Among the entire female group (Group 3), Border Collie was the largest population (*n* = 22, 23.2%) followed by Crossbreed (*n* = 15, 15.8%) and Labrador Retriever (*n* = 11, 11.6%). There was a significantly higher proportion of Border Collies in the entire female group (Group 3) compared with the adult-onset non-dioestrus group (Group 2) (Fisher’s exact test, *p* < 0.0001). In the logistic regression modelling, advancing age was associated with increasing odds of DM in the adult-onset non-dioestrus group (*p* value < 0.001, OR = 1.11, 95% CI = 1.09–1.13 per one-year increase in age). Breed was also strongly associated with the odds of DM (variable *p* value < 0.001).

When utilising the non-diabetic VetCompass population as controls, compared to Crossbreeds, the breeds at greatest odds of DM were the Samoyed (OR = 23.5, 95% CI = 14.2–39.0), Tibetan Terrier (OR = 11.1, 95% CI = 7.33–16.7) and Dachshund (OR = 10.9, 95% CI = 6.5–18.1) (Fig. 6, Supplementary Table 1). The Boxer, German Shepherd Dog, Pug and Cockapoo were the breeds at lowest odds of DM, however the OR could not be calculated by the model because there were no diabetic cases in these breeds recorded in the UKCDRA. The Labrador Retriever and Husky breeds had near neutral odds of DM compared to Crossbreeds (OR = 1.14, 95% CI = 0.89–1.5; OR = 0.89, 95% CI = 0.4–2.0 respectively).

In the entire female group (Group 3), breed was also significantly associated with the odds of DM (variable *p* value < 0.001). Compared to Crossbreeds, breeds at greatest DM odds in the entire female group were the Samoyed (OR = 118.7, 95% CI = 45.8–307.5), Border Collie (OR = 10.9, 95% CI = 5.7–21.0) and Husky (OR = 9.1, 95%CI = 3.3–25.0), although these results must be treated with caution as the control group contained male and female dogs of varying neuter status. Several of the breeds included in the analysis had no female entire cases submitted to the UKCDRA and therefore a precise OR for DM in female entire dogs of these breeds could not be calculated.

## Discussion

This study presents data relating to submissions to the UK Canine Diabetes Register and Archive (UKCDRA) more than 20 years after its initiation, providing an update to the initial report in 2003 [4], identifying trends and highlighting clinically relevant information for practising veterinarians. Because registries contain observational data, with minimal inclusion or exclusion criteria, they can serve as useful research tools to identify risk factors and improve understanding of a disease [36] as well as providing clinical benchmarks [37]. When accompanied by archives of clinical samples, the potential research scope of disease registries is broadened even further. A total of 1,072 cases of canine DM, comprising juvenile and adult-onset forms, were analysed from data submitted between December 2005 and December 2019. Among the adult-onset DM groups, the mean age of DM diagnosis varied among breeds but no clear pattern of seasonality in the diagnosis of canine DM was identified. This study also identified 15 cases of juvenile canine DM submitted to the archive between 2005 and 2019, with Labrador Retrievers the most highly represented juvenile DM breed.

This study has also provided insights into management of canine diabetes in the UK over a 14 year period. In the non-dioestrus DM group, the total insulin dose per day was significantly lower in cases treated SID compared to BID. The odds ratio for development of DM among dog breeds broadly supported the breed risks identified in previous reports.

### Juvenile DM group

An interesting observation in this study is the documentation of a small number of UK juvenile DM cases and the high representation of Labrador Retrievers in this group, as has previously been reported [10]. Due to the very low frequency of juvenile cases among the canine DM population (15/1072 [1.4%] canine DM cases included in this study), these cases would be more difficult to collate without the existence of the UKCDRA, except as individual case reports. The early age of onset in these cases is comparable to the juvenile age of onset of monogenic forms of DM in humans, raising the possibility of a monogenic form of canine DM with early age of onset which is distinct from polygenic forms of DM which occur at older ages [10].

### Adult-onset DM groups

Of the 1057 adult-onset DM cases submitted between December 2005 and December 2019, 951 cases (90.0%) were in the non-dioestrus group (Group 2), comprising male entire, male neutered and female neutered dogs at the time of diagnosis, and 106 cases (10.0%) in the female entire group (Group 3). In contrast, a recent VetCompass study found that female entire dogs made up 18.2% of non-diabetic dogs and 19.1% of diabetic dogs in the UK VetCompass population in 2016 [7]. There is no clear reason for the difference between the UKCDRA diabetic population and the previously reported VetCompass population, but this may include changes in neutering practices with time, or recruitment bias, e.g. owners giving consent for their diabetic dog to participate in a clinical database and archive may also be more likely to neuter their pet.

### Age of onset and seasonality

The median age at first diagnosis was similar in both the non-dioestrus (Group 2) and female entire (Group 3) UKCDRA groups and these ages were similar to median age of diagnosis of canine DM reported previously [4, 6, 7]. However, when the mean age at diagnosis was examined for individual breeds with > 5 dogs in the non-dioestrus group, there was a significant difference among breeds. Post-hoc comparison identified five breeds with a significantly different mean age at diagnosis compared to Crossbreeds, all of which experienced earlier DM onset. This could be related to differences in the speed of aging and average life expectancy among breeds, it could represent differences in diabetes risk genetic burden across breeds, or could highlight differences in the aetiology of DM among dog breeds, as had been speculated elsewhere [11, 13, 29].

In the present study, there was no clear pattern of seasonality of diagnosis in Group 2, (comprising dogs who should were male, male neutered or female neutered at diagnosis), although the fewest cases were diagnosed in the month of December, contrasting with previous reports of a winter peak in diagnosis of canine diabetes [4, 38]. A recent study of seasonality in diabetes diagnosis in young people suggested a peak in January in type 1 (autoimmune) diabetes diagnosis and a peak in August in type 2 diabetes [39] with environmental factors such as hot or cold stress, viruses, vitamin D level and nutritional status among the factors proposed as affecting diabetes risk. It was not possible to examine environmental factors such as corticosteroid use in cases in this study [40], as this information was not provided on the UKCDRA submission form. Similar to the adult-onset non-dioestrus DM cases (Group 2), there was no clear pattern of seasonality of diagnosis in the female entire group (Group 3). Dogs are considered to be non-seasonally monoestrous, however there is conflicting evidence for seasonality in oestrus in dogs. The Basenji reportedly has annual oestrus cycles regulated by photoperiod [41] however, other studies contradict this [42] so the significance of this observation in the female entire DM group is not clear.

### Clinical management and monitoring

The study also revealed evolving clinical practices, particularly a transition from once-daily Insuvet lente to twice-daily Caninsulin administration since previous UKCDRA reports [4] reflective of changes in product availability. Notably the recommended starting dose for Caninsulin according to the data sheet is 0.5 to 1.0 iu/kg once daily in dogs. The daily insulin dose per kg was generally higher in dogs treated BID than those treated SID, for all insulin treatments, but the dose per kg per injection was lower in dogs treated BID. (Fig. S1 and S2). Previously available Insuvet products (Lente and PZI, Schering Plough Animal Health) were discontinued in 2010. Caninsulin (MSD Animal Health) has been available throughout the lifespan of the UKCDRA, however ProZinc (Boehringer Ingelheim Animal Health UK Ltd) became available in 2016 and was only more widely marketed for use in dogs in 2019.

This study was not designed to evaluate efficacy of insulin therapy in UK diabetic dogs, but rather to report on the treatment choices made by UK veterinarians. Nonetheless, fructosamine concentrations were evaluated, reflecting average glycaemic control over the preceding 1–2 weeks, in each dog. Fructosamine concentration was not significantly different between once-daily and twice-daily treated dogs. This was in contrast to the first UKCDRA study, where twice daily treatment was associated with improved markers of glycaemic control [4]. However due to differences between individual dogs, it should still be noted that fructosamine concentration is more appropriate for use longitudinally in a single patient rather than indicating glycaemic control at a population level. When daily insulin dose was compared to measures of glycaemic control, fructosamine concentration was significantly negatively correlated with insulin dose in the non-dioestrus group (Group 2 – including males, neutered males and neutered females), suggesting that a higher insulin dose (across all treatment types and dosing schedules) resulted in a lower fructosamine concentration. However, the same trend did not reach significance in the entire female group (Group 3), which may be related to the smaller group size. As has been reported previously [30, 43] there was also a positive correlation between HbA1c and fructosamine concentrations in both the non-dioestrus (Group 2) and female entire (Group 3) groups, which is not surprising as both parameters reflect average blood glucose.

The reporting of concurrent conditions in diabetic dogs was highly variable and therefore not reliable, but the conditions noted broadly reflected those reported in other canine diabetes studies [6, 22]. Future studies could be improved by collecting data specifically about the presence or absence of particular conditions of relevance e.g. pancreatitis, hypertriglyceridaemia, and identifying trends in concurrent disease among different breeds.

### Diabetic breeds in the UKCDRA compared to non-diabetic breeds in VetCompass

The results of the multivariable binary logistic regression analysis for cases of DM in the adult-onset non-dioestrus group (Group 2 – dogs who were male, male neutered or female neutered at diagnosis) compared to the 2016 VetCompass denominator population (with known or suspected diabetic dogs removed) largely supported the findings of previous analyses of breed risk of DM [2, 6, 7, 22]. The VetCompass 2016 denominator population was a useful validated resource for the calculation of breed risk of DM in this study, due to the very large number of clinical records available, and this was a larger breed comparator population than used in the previous study [4]. Ideally the control population in the current study should represent the population from which the diabetic cases were derived, however this exact population was not available, which is a limitation in this analysis. Direct comparison of the diabetic UKCDRA and non-diabetic VetCompass populations for the purpose of breed distribution must therefore be undertaken with caution, as the cases were not selected directly from the VetCompass population [33].

Attempts to make the control population as representative as possible of the population from which the cases were submitted, included the exclusion of potential and known diabetic cases and removal of dogs < 3 years old. Despite these measures, it is still possible that some of the dogs in the control group may have developed DM in future. However in general, breed-related findings do reflect previous studies of the breeds in the UKCDRA [4, 10]. Despite their relatively low numbers of cases in the archive, breeds with greatest odds for DM in the current study, as a result of being over-represented in the UKCDRA compared to VetCompass were the Samoyed (OR = 23.5;), Tibetan Terrier (OR = 11.1; 95% CI = 7.34–16.7), Dachshund (OR = 10.9; 95% CI = 6.5–18.1) and Cairn Terrier (OR = 8.3; 95% CI = 5.69–12.2). These were also the four breeds at greatest DM risk in a previous analysis of breeds in the UKCDRA [10]. The Cairn and Tibetan Terrier breeds were also reported to be at high risk in previous studies of the diabetic dogs within the VetCompass population itself [6] however, the Samoyed and Dachshund breeds were not reported in these studies.

When exploring population risk, it is also valuable to identify individuals at reduced risk of disease. Notably, the Boxer, German Shepherd Dog, Cockapoo and Pug all had no cases submitted to the UKCDRA, despite having > 5000 of each breed in the reference Vet Compass population. These breeds have all been reported to be at very low risk of DM in previous studies [6, 7, 22]. The Springer Spaniel (OR = 0.32; 95% CI = 0.16–0.64), Shih-tzu (OR = 0.19; 95% CI = 0.09–0.44) and Golden Retriever (OR = 0.55; 95% CI = 0.26–1.16) breeds, reported here as low risk of DM, have also previously been reported as such [2, 7, 22] and the Chihuahua (OR = 0.042; 95% CI = 0.006–0.30) has recently been reported to be at low risk by a separate study of diabetes within the UK VetCompass population [7]. The Labrador Retriever, one of the most popular breeds in the UK, and a highly represented breed in the UKCDRA, shared a similar risk of DM with Crossbreeds in the present study (OR = 1.14; 95% CI 0.89–1.47) and therefore the high number of Labrador retriever submissions to the archive more likely represents the popularity of the breed than a particularly high breed risk of DM.

Breed risk of DM among the adult-onset female entire group (Group 3) was remarkably similar to the findings in the non-dioestrus group, albeit with wider confidence intervals due to smaller sample numbers in these groups, and more breeds excluded from the analysis due to having no cases in the UKCDRA. This is a very interesting observation, although must be treated with caution since the VetCompass control population contained male and female dogs of varying neuter status, and varying age at neutering, whereas Group 3 contains only females reported to be entire at the time of DM diagnosis, so Odds Ratios could be influenced by factors such as different neutering practices among breeds. Nonetheless, finding similar breeds represented in both the dioestrus and non-dioestrus DM groups in the UKCDRA suggests that similar genetic factors may predispose to both types of DM in these breeds. These genes may impact beta cell survival, function or renewal under stress, potentially implicating beta cell health and resilience in canine DM risk, with dioestrus being only one of several potential ‘trigger’ factors in high risk breeds.

### Limitations of the present study and ongoing work

The UKCDRA contains a large volume of clinical data and samples which are valuable for research into canine DM. However, limitations of the study include variability in the completeness of clinical data submitted, reliance on practitioner-reported diagnoses, and inconsistencies in the reporting of co-morbidities and environmental factors such as corticosteroid use or dioestrus. When considering the inclusion of cases in the various analyses, juvenile cases (Group 1) and female entire cases (Group 3) were separated from the adult-onset non-dioestrus group (Group 2), this was done in an attempt to minimise the inclusion of phenocopies (individuals with the same disease phenotype but different underlying aetiology) in each analysis group. However, there is still much unknown about the aetiology of canine DM [1, 13, 14]. Therefore it is still possible that dogs with different types of DM pathogenesis were included in the same analysis group.

In addition, by nature of including only dogs < 1 year in the juvenile DM group and dogs > 3 years in the adult-onset groups, any dogs diagnosed with DM between 1 and 3 years were excluded from the analysis. Although there were only 19 such cases submitted between December 2005 and December 2019, they may also represent a relevant population for inclusion in future studies of canine DM and may have contributed further to the age-of-onset analysis had they been included.

Breed associations were made on a group by group basis, comparing the dogs in each diabetic group to the whole VetCompass population rather separating by sex or neuter status. This method was also used in the previous study in which comparison was made to breeds within an insurance database [4]. Although male dogs were included in the control group, it was considered unlikely to bias the results substantially, given the consistently similar distribution of male and female dogs within a range of breeds previously studied within VetCompass [44–47] and despite this limitation, it was reassuring that there was agreement in at-risk and low risk DM breeds with several other studies.

The final limitation in this analysis is that the VetCompass control population was taken from a single year (2016) whereas the UKCDRA breed data comprised data acquired between December 2005 and December 2019. Therefore, the year-on-year changes in breed popularity in the UK which may be reflected in the UKCRDA sample submissions would not be fully reflected in the 2016 VetCompass breed data. Furthermore, Crossbreeds were all considered together in the current analysis, even though they are not a homogeneous population and certain breed crosses might be much more common in this population than others.

In summary, the UKCDRA is a valuable source of clinical data and blood samples for the study of canine DM and reference databases such as VetCompass are pivotal in providing control populations for study of disease. The primary findings of this study support previous observations about the epidemiology of the UK canine diabetic population and the breeds at greatest and lowest risk of DM, as well as highlighting the differing age of onset between breeds and the need for further study of the aetiology and risk factors for canine DM in particular breeds. This study has also informed a new online questionnaire design for future submissions to the UKCDRA, requesting more detailed information about patients, including environmental factors in the period prior to DM diagnosis, and co-morbidities.

## Supplementary Information

Below is the link to the electronic supplementary material.

**Supplementary Material 1**: **Supplementary Fig. S1**: The proportion of each insulin treatment recorded in each year since initiation of the UKCDRA, including all adult-onset cases of DM.

**Supplementary Material 2**: **Supplementary Fig. S2**: The proportion of diabetic cases in the UKCDRA treated with insulin once a day (SID) or twice a day (BID) in each year since initiation of the UKCDRA, including all adult-onset cases of DM.

**Supplementary Material 3**: **Supplementary Table S1**: Breed risk for DM in adult-onset non-dioestrus DM group (*n* = 873) calculated by reference to VetCompass denominator population (*n* = 455335). Breeds ordered according to odds ratio for DM. CI: confidence interval. **Supplementary Table S2**: Breed risk for DM in entire female DM group (*n* = 95), calculated by reference to VetCompass denominator population (*n* = 455335). Breeds ordered according to odds ratio for DM. CI: confidence interval.

## Data Availability

Data is provided within the manuscript. Further details and access to relevant VetCompass data are available by contacting the corresponding author.

## Footnotes

1
https://venomcoding.org/.
