# Supplementary material for: Epidemiology and clinical management of 1072 dogs with diabetes mellitus in a UK diabetes register
Source: Companion Anim Health Genet. 2025 Oct 10;12:7. doi: 10.1186/s40575-025-00146-x (PMC12512512; doi:10.1186/s40575-025-00146-x)
Supplement: Supplementary file 3 — Supplementary Material 3: Supplementary Table S1: Breed risk for DM in adult-onset non-dioestrus DM group (n = 873) calculated by reference to VetCompass denominator population (n = 455335). Breeds ordered according to odds ratio for DM. CI: confidence interval. Supplementary Table S2: Breed risk for DM in entire female DM group (n = 95), calculated by reference to VetCompass denominator population (n = 455335). Breeds ordered according to odds ratio for DM. CI: confidence interval. [file 40575_2025_146_MOESM3_ESM.docx]

**Supplementary Table S1:** Breed risk for DM in adult-onset non-dioestrus DM group (n = 873) calculated by reference to VetCompass denominator population (n = 455335). Breeds ordered according to odds ratio for DM. CI: confidence interval.

| **Breed** | **VetCompass** | | **Diabetes Register** | | **Total** | **P value** | **Odds Ratio** | **95% CI** |
| --- | --- | --- | --- | --- | --- | --- | --- | --- |
|  | **n** | **%** | **n** | **%** |  |  |  |  |
| Samoyed | 407 | 0.09% | 17 | 1.95% | 424 | <.001 | 23.5 | 14.2 - 39.0 |
| Tibetan Terrier | 1269 | 0.28% | 26 | 2.98% | 1295 | <.001 | 11.1 | 7.3 - 16.7 |
| Dachshund | 855 | 0.19% | 16 | 1.83% | 871 | <.001 | 10.9 | 6.5 - 18.1 |
| Cairn Terrier | 1929 | 0.42% | 31 | 3.55% | 1960 | <.001 | 8.31 | 5.7 - 12.2 |
| Standard Doberman Pinscher | 1481 | 0.33% | 15 | 1.72% | 1496 | <.001 | 6.14 | 3.6 - 10.4 |
| Scottish Terrier | 1082 | 0.24% | 11 | 1.26% | 1093 | <.001 | 5.48 | 3.0 - 10.1 |
| Miniature Schnauzer | 5148 | 1.13% | 36 | 4.12% | 5184 | <.001 | 4.24 | 3.0 - 6.0 |
| West Highland White Terrier | 14855 | 3.26% | 95 | 10.88% | 14950 | <.001 | 3.20 | 2.5 - 4.1 |
| Border Terrier | 6860 | 1.51% | 29 | 3.32% | 6889 | <.001 | 2.35 | 1.6 - 3.5 |
| Cocker Spaniel | 20583 | 4.52% | 52 | 5.96% | 20635 | 0.01 | 1.49 | 1.1 - 2.0 |
| Rottweiler | 4620 | 1.01% | 11 | 1.26% | 4631 | 0.234 | 1.45 | 0.8 - 2.6 |
| Border Collie | 16248 | 3.57% | 42 | 4.81% | 16290 | 0.062 | 1.37 | 0.98 - 1.9 |
| Cavalier King Charles Spaniel | 12445 | 2.73% | 27 | 3.09% | 12472 | 0.229 | 1.28 | 0.9 - 1.9 |
| Bichon Frise | 8760 | 1.92% | 18 | 2.06% | 8778 | 0.341 | 1.26 | 0.8 - 2.0 |
| Yorkshire Terrier | 19305 | 4.24% | 45 | 5.15% | 19350 | 0.175 | 1.25 | 0.9 - 1.7 |
| Labrador Retriever | 40733 | 8.95% | 83 | 9.51% | 40816 | 0.304 | 1.14 | 0.9 - 1.5 |
| **Crossbreed** | 120776 | 26.52% | 215 | 24.63% |  |  | Base | - |
| Husky | 4441 | 0.98% | 6 | 0.69% | 4447 | 0.777 | 0.89 | 0.4 - 2.0 |
| Toy/Miniature Poodle | 3874 | 0.85% | 6 | 0.69% | 3880 | 0.722 | 0.86 | 0.4 - 1.9 |
| Jack Russell Terrier | 36046 | 7.92% | 49 | 5.61% | 36095 | 0.03 | 0.71 | 0.5 - 0.97 |
| Lhasa Apso | 8398 | 1.84% | 10 | 1.15% | 8408 | 0.253 | 0.69 | 0.4 - 1.3 |
| Golden Retriever | 6837 | 1.50% | 7 | 0.80% | 6844 | 0.116 | 0.55 | 0.3 - 1.2 |
| Springer Spaniel | 14058 | 3.09% | 8 | 0.92% | 14066 | 0.001 | 0.32 | 0.2 - 0.6 |
| Shih-tzu | 19230 | 4.22% | 6 | 0.69% | 19236 | <.001 | 0.19 | 0.1 - 0.4 |
| Staffordshire Bull Terrier | 37127 | 8.15% | 11 | 1.26% | 37138 | <.001 | 0.16 | 0.1 - 0.3 |
| Chihuahua | 16405 | 3.60% | 1 | 0.11% | 16406 | 0.002 | 0.04 | 0.01 - 0.3 |
| Boxer | 6614 | 1.45% | 0 | 0.00% | 6614 | - | - | - |
| Cockapoo | 5178 | 1.14% | 0 | 0.00% | 5178 | - | - | - |
| German Shepherd Dog | 13249 | 2.91% | 0 | 0.00% | 13249 | - | - | - |
| Pug | 6522 | 1.43% | 0 | 0.00% | 6522 | - | - | - |
| Total | 455335 | 1 | 873 | 1 |  |  |  |  |

**Supplementary Table S2:** Breed risk for DM in entire female DM group (n = 95), calculated by reference to VetCompass denominator population (n = 455335). Breeds ordered according to odds ratio for DM. CI: confidence interval.

|  | **VetCompass** | | **Diabetes Register** | | **Total** | **P value** | **Odds Ratio** | **95% CI** |
| --- | --- | --- | --- | --- | --- | --- | --- | --- |
|  | **n** | **%** | **n** | **%** |  |  |  |  |
| Samoyed | 407 | 0.1% | 6 | 6.3% | 413 | <.001 | 119 | 45.8 - 307.5 |
| Border Collie | 16248 | 3.6% | 22 | 23.2% | 16270 | <.001 | 10.9 | 5.7 - 21.0 |
| Husky | 4441 | 1.0% | 5 | 5.3% | 4446 | <.001 | 9.07 | 3.3 – 25.0 |
| Cairn Terrier | 1929 | 0.4% | 2 | 2.1% | 1931 | 0.005 | 8.35 | 1.9 - 36.5 |
| Tibetan Terrier | 1269 | 0.3% | 1 | 1.1% | 1270 | 0.074 | 6.35 | 0.84 - 48.0 |
| Standard Doberman Pinscher | 1481 | 0.3% | 1 | 1.1% | 1482 | 1.01E-01 | 5.44 | 0.72 - 41.2 |
| Miniature Schnauzer | 5148 | 1.1% | 2 | 2.1% | 5150 | 0.13 | 3.13 | 0.72 - 13.7 |
| German Shepherd Dog | 13249 | 2.9% | 5 | 5.3% | 13254 | 0.031 | 3.04 | 1.1 - 8.4 |
| Yorkshire Terrier | 19305 | 4.2% | 7 | 7.4% | 19312 | 0.019 | 2.92 | 1.2 - 7.2 |
| West Highland White Terrier | 14855 | 3.3% | 5 | 5.3% | 14860 | 0.054 | 2.71 | 0.99 - 7.5 |
| Border Terrier | 6860 | 1.5% | 2 | 2.1% | 6862 | 0.257 | 2.35 | 0.5 - 10.3 |
| Labrador Retriever | 40733 | 8.9% | 11 | 11.6% | 40744 | 0.05 | 2.17 | 0.99 - 4.7 |
| Cocker Spaniel | 20583 | 4.5% | 3 | 3.2% | 20586 | 0.8 | 1.17 | 0.3 - 4.1 |
| **Crossbreed** | 120776 | 26.5% | 15 | 15.8% | 120791 |  | Base |  |
| Bichon Frise | 8760 | 1.9% | 1 | 1.1% | 8761 | 0.935 | 0.92 | 0.1 – 7.0 |
| Jack Russell Terrier | 36046 | 7.9% | 4 | 4.2% | 36050 | 0.841 | 0.89 | 0.3 - 2.7 |
| Cavalier King Charles Spaniel | 12445 | 2.7% | 1 | 1.1% | 12446 | 6.73E-01 | 0.65 | 0.1 - 4.9 |
| Springer Spaniel | 14058 | 3.1% | 2 | 2.1% | 14060 | 0.98 | - | - |
| Boxer | 6614 | 1.5% | 0 | 0.0% | 6614 | - | - | - |
| Chihuahua | 16405 | 3.6% | 0 | 0.0% | 16405 | - | - | - |
| Cockapoo | 5178 | 1.1% | 0 | 0.0% | 5178 | - | - | - |
| Dachshund | 855 | 0.2% | 0 | 0.0% | 855 | - | - | - |
| Golden Retriever | 6837 | 1.5% | 0 | 0.0% | 6837 | - | - | - |
| Lhasa Apso | 8398 | 1.8% | 0 | 0.0% | 8398 | - | - | - |
| Toy/Miniature Poodle | 3874 | 0.9% | 0 | 0.0% | 3874 | - | - | - |
| Pug | 6522 | 1.4% | 0 | 0.0% | 6522 | - | - | - |
| Rottweiler | 4620 | 1.0% | 0 | 0.0% | 4620 | - | - | - |
| Staffordshire Bull Terrier | 37127 | 8.2% | 0 | 0.0% | 37127 | - | - | - |
| Shih-tzu | 19230 | 4.2% | 0 | 0.0% | 19230 | - | - | - |
| Scottish Terrier | 1082 | 0.2% | 0 | 0.0% | 1082 | - | - | - |
| Total | 455335 |  | 95 |  | 455430 |  |  |  |
